# (1*RS*,6*SR*)-Ethyl 4-(2,4-dichloro­phen­yl)-6-(4-fluoro­phen­yl)-2-oxocyclo­hex-3-ene-1-carboxyl­ate

**DOI:** 10.1107/S160053681100184X

**Published:** 2011-01-22

**Authors:** Grzegorz Dutkiewicz, B. Narayana, K. Veena, H. S. Yathirajan, Maciej Kubicki

**Affiliations:** a Department of Chemistry, Adam Mickiewicz University, Grunwaldzka 6, 60-780 Poznań, Poland; b Department of Studies in Chemistry, Mangalore University, Mangalagangotri 574 199, India; c Department of Studies in Chemistry, University of Mysore, Manasagangotri, Mysore 570 006, India

## Abstract

There are two symmetry-independent mol­ecules in the asymmetric unit of the title compound, C_21_H_17_Cl_2_FO_3_. Both these mol­ecules are very similar: the normal probability plots for bond lengths, angles and even for torsion angles show that the differences are of a statistical nature. A pseudocentre of symmetry is located between the symmetry-independent mol­ecules at [0.245 (1), 0.535 (19), 0.909 (1)]. The cyclo­hexene rings have slightly distorted sofa conformations in both mol­ecules and the two benzene rings are inclined by dihedral angles of 61.33 (14) and 62.85 (14)°. In the crystal, relatively short inter­molecular C—H⋯O inter­actions join mol­ecules into homomolecular (*i.e.* ⋯*AAA*⋯ and ⋯*BBB*⋯) chains along the *b* axis. These chains are inter­connected by further heteromolecular C—H⋯O inter­actions.

## Related literature

For normal probability plots, see: Abrahams & Keve (1971 ▶). For asymmetry parameters, see: Duax & Norton (1975 ▶). For similar structures, see: Anuradha *et al.* (2009 ▶); Li *et al.* (2009 ▶); Fun *et al.* (2008 ▶, 2009 ▶, 2010 ▶); Badshah *et al.* (2009 ▶), Dutkiewicz *et al.* (2011*a* ▶,*b* ▶). For a description of the Cambridge Structural Database, see: Allen (2002 ▶).
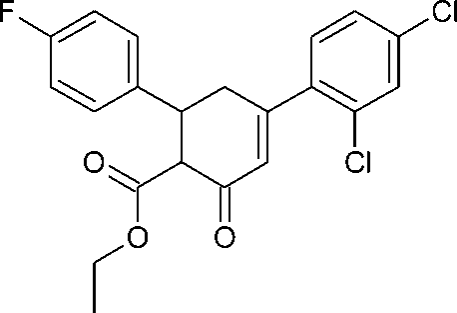


## Experimental

### 

#### Crystal data

C_21_H_17_Cl_2_FO_3_
*M* *_r_* = 407.25
Orthorhombic, 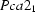

*a* = 32.321 (5) Å
*b* = 5.437 (2) Å
*c* = 22.309 (3) Å
*V* = 3920.3 (17) Å^3^
*Z* = 8
Mo *K*α radiation
μ = 0.36 mm^−1^
*T* = 295 K
0.35 × 0.3 × 0.2 mm

#### Data collection

Oxford Diffraction Xcalibur Eos diffractometer
Absorption correction: multi-scan (*CrysAlis PRO*; Oxford Diffraction, 2009 ▶) *T*_min_ = 0.819, *T*_max_ = 1.000
7901 measured reflections
4968 independent reflections
3654 reflections with *I* > 2σ(*I*)
*R*_int_ = 0.022

#### Refinement

*R*[*F* ^2^ > 2σ(*F* ^2^)] = 0.039
*wR*(*F* ^2^) = 0.104
*S* = 1.01
4968 reflections
487 parameters
1 restraint
H-atom parameters constrained
Δρ_max_ = 0.17 e Å^−3^
Δρ_min_ = −0.22 e Å^−3^
Absolute structure: Flack (1983 ▶), 623 Friedel pairs
Flack parameter: 0.03 (6)

### 

Data collection: *CrysAlis PRO* (Oxford Diffraction, 2009 ▶); cell refinement: *CrysAlis PRO*; data reduction: *CrysAlis PRO*; program(s) used to solve structure: *SIR92* (Altomare *et al.*, 1993 ▶); program(s) used to refine structure: *SHELXL97* (Sheldrick, 2008 ▶); molecular graphics: *SHELXTL* (Sheldrick, 2008 ▶) and *Mercury* (Macrae *et al.*, 2008 ▶); software used to prepare material for publication: *SHELXL97*.

## Supplementary Material

Crystal structure: contains datablocks I, global. DOI: 10.1107/S160053681100184X/dn2651sup1.cif

Structure factors: contains datablocks I. DOI: 10.1107/S160053681100184X/dn2651Isup2.hkl

Additional supplementary materials:  crystallographic information; 3D view; checkCIF report

## Figures and Tables

**Table 1:** Hydrogen-bond geometry (Å, °)

*D*—H⋯*A*	*D*—H	H⋯*A*	*D*⋯*A*	*D*—H⋯*A*
C1*A*—H1*A*⋯O12*A*^i^	0.98	2.40	3.306 (4)	154
C62*A*—H62*A*⋯O12*A*^i^	0.93	2.55	3.442 (5)	162
C1*B*—H1*B*⋯O12*B*^ii^	0.98	2.39	3.292 (4)	153
C62*B*—H62*B*⋯O12*B*^ii^	0.93	2.47	3.358 (4)	161
C14*A*—H14*A*⋯Cl1^iii^	0.97	2.82	3.725 (6)	155
C3*A*—H3*A*⋯O2*B*	0.93	2.54	3.360 (5)	148
C3*B*—H3*B*⋯O2*A*	0.93	2.51	3.352 (5)	151

Symmetry codes: (i) 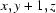; (ii) 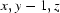; (iii) 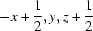.

**Fig. 1.**
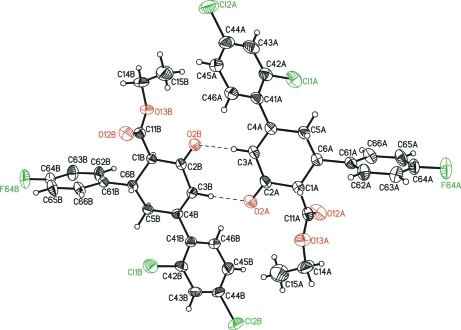
Anisotropic ellipsoid representation of the molecules of I together with atom labelling scheme. The ellipsoids are drawn at 33% probability level, hydrogen atoms are depicted as spheres with arbitrary radii, weak C-H···O hydrogen bonds are shown as dashed lines.

**Fig. 2.**
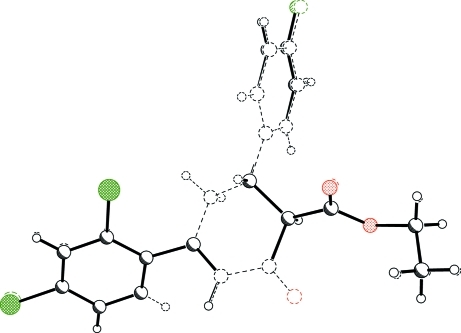
The comparison of two symmetry-independent molecules of I. The cyclohexene rings were fitted onto each other.

**Fig. 3.**
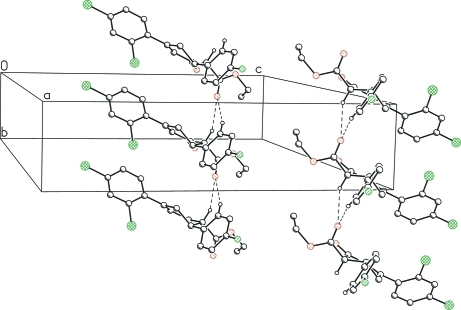
The hydrogen-bonded homomolecular chains along y-direction. Hydrogen bonds are shown as dashed lines.

**Fig. 4.**
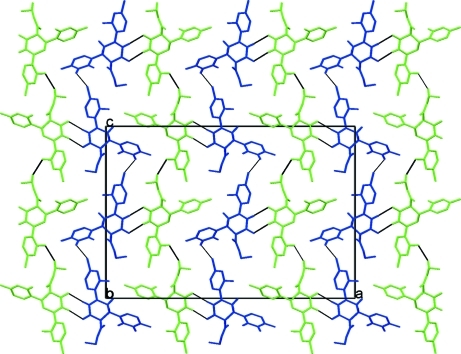
The crystal packing as seen along [010] direction; hydrogen bonds are shown as dashed lines. The symmetry-independent molecules are drawn with different colours.

## supplementary crystallographic information

### Comment

In the course of our studies on chalcone derivatives (Dutkiewicz *et al.*, 2011*a*,*b*), we have determined the crystal structure of ethyl 4-(2,4-dichlorophenyl)-6-(4-fluorophenyl)-2-oxocyclohex-3-ene-1-carboxylate (I, Scheme 1).

There are two symmetry-independent molecules in the asymmetric part of the unit cell(Fig. 1). Interestingly enough, it is quite common among similar structures. Out of eight structures of 4,6-diaryl derivatives of 2-oxocyclohex-3-ene found in the CSD (Allen, 2002), 3 have *Z*'=2 (Fun *et al.*, 2008, 2009, 2010). In I the two independent molecules are very similar (Fig. 2); normal probability plot calculations (Abrahams & Keve, 1971) show very high correlation between experimental and theoretical normal distribution of the differences between bond lengths (*R*^2^=0.96), bond angles (*R*^2^=0.98) and even between the torsion angles (*R*^2^=0.96). The pseudo-centre of symmetry can be found between the symmetry independent molecules. The sums of appropriate coordinates are similar and the esd's of such found point are quite good: <*x_i_*A+*x*_i_B>=0.245 (1), <*y_i_*A+*y_i_*B>=0.535 (19), <*z_i_*A+*z_i_*C>=0.909 (1). Since these molecules are so similar, we will analyze one of them and the numeric values for the other will be given in square brackets.

The cyclohexene rings adopt slightly distorted sofa conformation (Fig. 2), the asymmetry parameter Δ*C_s_*^3^ (Duax & Norton, 1975) is 3.26° [3.60°]. In this ring five atoms C1 - C5 are almost coplanar, maximum deviation from the least-squares plane is 0.028 (3)Å [0.024 (3) Å], while the sixth atom, C6, is significantly out of this plane, by 0.672 (5)Å [0.685 (4) Å]. Similar conformation was found in related compound (Anuradha *et al.*, 2009; Li *et al.*, 2009; Fun *et al.*, 2008, 2009, 2010; Badshah *et al.*, 2009; Dutkiewicz *et al.*, 2011*a*,*b*).

The overall conformation of a molecule I (*cf.* Fig. 1) can be characterized by the dihedral angles between the phenyl rings, of 61.33 (14)° [62.85 (14)°}, and between these rings and the mean plane of the cyclohexene ring which are equal to 63.41 (13)° [64.00 (12)°] for the dichlorophenyl and 84.85 (12)° [85.07 (12)°] for fluorophenyl ring. In the crystal of the methyl analogue, methyl 4,6-bis(4-fluorophenyl)-2-oxocyclohex- 3-ene-1-carboxylate (Fun *et al.*, 2010) there are two symmetry independent molecules, but the overall conformation of both of them is similar to that of I. It might be noted that generally for similar structures the angles between the cyclohexene plane and phenyl ring at position 4 were smaller, not only because of the lack of the steric hindrance but even for the two *ortho*-substituted rings: in 3-(2-hydroxyphenyl)-5-phenyl-6-ethoxycarbonylcyclohex-2-enone (refcode QESTEO) it is 18.1°, and the same value it has been fund in 3-(2-hydroxyphenyl)-5-(2-methylphenyl)-6-ethoxycarbonyl-2-cyclohexen-one (NAMKES).

In the crystal structure relatively short and directional C1—H1···O12(*x,y + 1,z*) and C62—H62···O12(*x,y + 1,z*) create homomolecular (*i.e.* ···AAA··· and ···BBB···) chains of molecules along *y*-directions (Fig. 3). These chains are interconnected by means of weaker but still directional C3—H3···O2 heteromolecular hydrogen bonds into three dimensional structure (Fig. 4).

### Experimental

A mixture of (2*E*)-1-(2,4-dichlorophenyl)-3-(4-fluorophenyl)prop-2-en-1-one (0.01 mol) and ethyl acetoacetate (0.01 mol) were refluxed for 2 hr in 10–15 ml of ethanol in the presence of 0.8 ml 10% NaOH. The crystals were obtained by a slow evaporation from toluene/cyclohexenone solution. C_21_H_17_Cl_2_FO_3_, C: 61.88(61.93%); H: 4.18(4.21%); m.p 381 K.

### Refinement

Hydrogen atoms were located geometrically (*C*(methyl)-H 0.96 Å, C(CH_2_)-H 0.97 Å, C(CH)—H 0.98 Å, C(arom)-H 0.93 Å) and refined as a riding model; the *U*_iso_ values of H atoms were set at 1.2 (1.5 for CH_3_ group) times *U*_eq_ of their carrier atom.

### Crystal data

**Table:**

C_21_H_17_Cl_2_FO_3_	*F*(000) = 1680
*M**_r_* = 407.25	*D*_x_ = 1.380 Mg m^−^^3^
Orthorhombic, *P**c**a*2_1_	Mo *K*α radiation, λ = 0.71073 Å
Hall symbol: P 2c -2ac	Cell parameters from 3531 reflections
*a* = 32.321 (5) Å	θ = 2.8–26.9°
*b* = 5.437 (2) Å	µ = 0.36 mm^−^^1^
*c* = 22.309 (3) Å	*T* = 295 K
*V* = 3920.3 (17) Å^3^	Prism, colourless
*Z* = 8	0.35 × 0.3 × 0.2 mm

### Data collection

**Table:**

Oxford Diffraction Xcalibur Eos diffractometer	4968 independent reflections
Radiation source: Enhance (Mo) X-ray Source	3654 reflections with *I* > 2σ(*I*)
graphite	*R*_int_ = 0.022
Detector resolution: 16.1544 pixels mm^-1^	θ_max_ = 26.9°, θ_min_ = 3.1°
ω scans	*h* = −40→26
Absorption correction: multi-scan (*CrysAlis PRO*; Oxford Diffraction, 2009)	*k* = −6→5
*T*_min_ = 0.819, *T*_max_ = 1.000	*l* = −10→28
7901 measured reflections	

### Refinement

**Table:**

Refinement on *F*^2^	Secondary atom site location: difference Fourier map
Least-squares matrix: full	Hydrogen site location: inferred from neighbouring sites
*R*[*F*^2^ > 2σ(*F*^2^)] = 0.039	H-atom parameters constrained
*wR*(*F*^2^) = 0.104	*w* = 1/[σ^2^(*F*_o_^2^) + (0.0609*P*)^2^ + 0.1808*P*] where *P* = (*F*_o_^2^ + 2*F*_c_^2^)/3
*S* = 1.01	(Δ/σ)_max_ < 0.001
4968 reflections	Δρ_max_ = 0.17 e Å^−^^3^
487 parameters	Δρ_min_ = −0.22 e Å^−^^3^
1 restraint	Absolute structure: Flack (1983), 623 Friedel pairs
Primary atom site location: structure-invariant direct methods	Flack parameter: 0.03 (6)

### Special details

**Table:**

Geometry. All s.u.'s (except the s.u. in the dihedral angle between two l.s. planes) are estimated using the full covariance matrix. The cell s.u.'s are taken into account individually in the estimation of s.u.'s in distances, angles and torsion angles; correlations between s.u.'s in cell parameters are only used when they are defined by crystal symmetry. An approximate (isotropic) treatment of cell s.u.'s is used for estimating s.u.'s involving l.s. planes.
Refinement. Refinement of *F*^2^ against ALL reflections. The weighted *R*-factor *wR* and goodness of fit *S* are based on *F*^2^, conventional *R*-factors *R* are based on *F*, with *F* set to zero for negative *F*^2^. The threshold expression of *F*^2^ > σ(*F*^2^) is used only for calculating *R*-factors(gt) *etc*. and is not relevant to the choice of reflections for refinement. *R*-factors based on *F*^2^ are statistically about twice as large as those based on *F*, and *R*- factors based on ALL data will be even larger.

### Fractional atomic coordinates and isotropic or equivalent isotropic displacement parameters (Å^2^)

**Table:**

	*x*	*y*	*z*	*U*_iso_*/*U*_eq_	
C1A	0.22407 (10)	0.3769 (5)	0.53563 (17)	0.0409 (9)	
H1A	0.2266	0.5312	0.5580	0.049*	
C11A	0.22528 (11)	0.1675 (6)	0.5794 (2)	0.0463 (9)	
O12A	0.23719 (10)	−0.0345 (5)	0.56771 (18)	0.0785 (11)	
O13A	0.21037 (9)	0.2333 (5)	0.63242 (13)	0.0648 (8)	
C14A	0.20292 (17)	0.0368 (10)	0.6764 (3)	0.0859 (17)	
H14B	0.2234	−0.0916	0.6711	0.103*	
H14A	0.2060	0.1029	0.7165	0.103*	
C15A	0.1625 (2)	−0.0664 (13)	0.6699 (4)	0.136 (3)	
H15C	0.1587	−0.1952	0.6988	0.203*	
H15B	0.1594	−0.1324	0.6302	0.203*	
H15A	0.1421	0.0593	0.6763	0.203*	
C2A	0.18159 (11)	0.3711 (7)	0.5059 (2)	0.0497 (10)	
O2A	0.15239 (8)	0.2667 (7)	0.52946 (16)	0.0846 (10)	
C3A	0.17750 (12)	0.4878 (7)	0.4485 (2)	0.0516 (11)	
H3A	0.1513	0.4964	0.4313	0.062*	
C4A	0.20962 (11)	0.5856 (6)	0.41828 (19)	0.0447 (9)	
C41A	0.20272 (11)	0.7015 (7)	0.35944 (19)	0.0477 (9)	
C42A	0.22106 (12)	0.6212 (7)	0.3069 (2)	0.0540 (10)	
Cl1	0.25473 (4)	0.3717 (2)	0.30848 (6)	0.0799 (4)	
C43A	0.21185 (15)	0.7226 (9)	0.2522 (2)	0.0673 (12)	
H43A	0.2239	0.6621	0.2173	0.081*	
C44A	0.18395 (15)	0.9189 (10)	0.2500 (2)	0.0700 (13)	
Cl2	0.17416 (5)	1.0573 (3)	0.18186 (7)	0.1044 (5)	
C45A	0.16521 (15)	1.0065 (8)	0.3010 (3)	0.0633 (13)	
H45A	0.1469	1.1383	0.2991	0.076*	
C46A	0.17414 (13)	0.8947 (7)	0.3547 (2)	0.0566 (11)	
H46A	0.1607	0.9491	0.3891	0.068*	
C5A	0.25213 (12)	0.5828 (7)	0.44552 (19)	0.0474 (10)	
H5A2	0.2567	0.7363	0.4667	0.057*	
H5A1	0.2725	0.5716	0.4138	0.057*	
C6A	0.25821 (10)	0.3685 (6)	0.48896 (18)	0.0418 (9)	
H6A	0.2546	0.2163	0.4660	0.050*	
C61A	0.30183 (10)	0.3660 (6)	0.51423 (19)	0.0425 (9)	
C62A	0.31576 (12)	0.5423 (7)	0.5529 (2)	0.0570 (12)	
H62A	0.2978	0.6665	0.5651	0.068*	
C63A	0.35596 (13)	0.5401 (8)	0.5743 (2)	0.0620 (13)	
H63A	0.3649	0.6597	0.6012	0.074*	
C64A	0.38220 (12)	0.3607 (8)	0.5556 (2)	0.0627 (12)	
F64A	0.42207 (8)	0.3609 (5)	0.57554 (17)	0.0988 (10)	
C65A	0.37003 (12)	0.1817 (7)	0.5177 (3)	0.0659 (13)	
H65A	0.3883	0.0587	0.5058	0.079*	
C66A	0.32956 (12)	0.1856 (6)	0.4969 (2)	0.0564 (12)	
H66A	0.3209	0.0637	0.4705	0.068*	
C1B	0.02150 (9)	0.1586 (5)	0.37208 (15)	0.0346 (8)	
H1B	0.0196	0.0045	0.3495	0.042*	
C11B	0.01996 (11)	0.3699 (6)	0.32869 (18)	0.0408 (9)	
O12B	0.00830 (9)	0.5711 (4)	0.34190 (15)	0.0625 (8)	
O13B	0.03490 (8)	0.3092 (4)	0.27537 (12)	0.0524 (7)	
C14B	0.04194 (15)	0.5093 (9)	0.2334 (2)	0.0734 (15)	
H14D	0.0204	0.6317	0.2383	0.088*	
H14C	0.0402	0.4463	0.1928	0.088*	
C15B	0.08209 (16)	0.6252 (11)	0.2421 (3)	0.106 (2)	
H15F	0.0853	0.7570	0.2139	0.159*	
H15E	0.1036	0.5058	0.2360	0.159*	
H15D	0.0838	0.6891	0.2821	0.159*	
C2B	0.06349 (11)	0.1693 (7)	0.40262 (19)	0.0467 (9)	
O2B	0.09266 (8)	0.2748 (7)	0.37863 (15)	0.0812 (10)	
C3B	0.06712 (12)	0.0550 (7)	0.4612 (2)	0.0485 (10)	
H3B	0.0930	0.0522	0.4795	0.058*	
C4B	0.03570 (11)	−0.0449 (6)	0.48991 (17)	0.0397 (9)	
C41B	0.04318 (11)	−0.1577 (6)	0.54973 (17)	0.0433 (9)	
C42B	0.02475 (12)	−0.0741 (7)	0.60187 (19)	0.0480 (9)	
Cl3	−0.01039 (4)	0.1688 (2)	0.59999 (5)	0.0698 (3)	
C43B	0.03397 (13)	−0.1748 (8)	0.65697 (19)	0.0594 (11)	
H43B	0.0214	−0.1154	0.6916	0.071*	
C44B	0.06171 (13)	−0.3624 (8)	0.65981 (19)	0.0614 (12)	
Cl4	0.07341 (5)	−0.4881 (4)	0.72951 (7)	0.1039 (6)	
C45B	0.08083 (14)	−0.4530 (8)	0.6099 (3)	0.0662 (14)	
H45B	0.0996	−0.5819	0.6128	0.079*	
C46B	0.07155 (13)	−0.3481 (7)	0.55472 (19)	0.0529 (10)	
H46B	0.0846	−0.4067	0.5204	0.063*	
C5B	−0.00690 (11)	−0.0492 (6)	0.46257 (18)	0.0399 (9)	
H5B2	−0.0274	−0.0407	0.4942	0.048*	
H5B1	−0.0108	−0.2034	0.4414	0.048*	
C6B	−0.01359 (10)	0.1638 (6)	0.41921 (17)	0.0362 (8)	
H6B	−0.0108	0.3168	0.4421	0.043*	
C61B	−0.05667 (10)	0.1587 (5)	0.39310 (17)	0.0358 (8)	
C62B	−0.06974 (12)	−0.0247 (7)	0.3548 (2)	0.0532 (12)	
H62B	−0.0509	−0.1425	0.3419	0.064*	
C63B	−0.11069 (13)	−0.0373 (8)	0.3350 (3)	0.0627 (14)	
H63B	−0.1193	−0.1647	0.3102	0.075*	
C64B	−0.13764 (11)	0.1402 (7)	0.3528 (2)	0.0561 (11)	
F64B	−0.17754 (7)	0.1264 (5)	0.33395 (15)	0.0911 (10)	
C65B	−0.12601 (12)	0.3256 (7)	0.3897 (2)	0.0640 (12)	
H65B	−0.1449	0.4450	0.4015	0.077*	
C66B	−0.08547 (11)	0.3338 (7)	0.4094 (2)	0.0525 (10)	
H66B	−0.0774	0.4616	0.4345	0.063*	

### Atomic displacement parameters (Å^2^)

**Table:**

	*U*^11^	*U*^22^	*U*^33^	*U*^12^	*U*^13^	*U*^23^
C1A	0.0400 (19)	0.0308 (16)	0.052 (2)	−0.0004 (15)	0.0038 (19)	−0.0023 (16)
C11A	0.038 (2)	0.0391 (19)	0.062 (3)	−0.0008 (16)	0.000 (2)	−0.0020 (19)
O12A	0.089 (2)	0.0401 (14)	0.107 (3)	0.0084 (15)	0.029 (2)	0.0090 (17)
O13A	0.082 (2)	0.0605 (17)	0.0519 (19)	−0.0128 (16)	0.0012 (16)	0.0016 (15)
C14A	0.095 (4)	0.103 (4)	0.060 (4)	−0.021 (3)	−0.013 (3)	0.031 (3)
C15A	0.103 (5)	0.167 (6)	0.137 (7)	−0.047 (5)	0.005 (5)	0.077 (6)
C2A	0.0354 (19)	0.058 (2)	0.056 (3)	−0.0026 (18)	0.008 (2)	0.004 (2)
O2A	0.0455 (16)	0.135 (3)	0.074 (2)	−0.0228 (18)	0.0031 (17)	0.035 (2)
C3A	0.0313 (19)	0.066 (2)	0.057 (3)	−0.0032 (18)	0.005 (2)	0.002 (2)
C4A	0.040 (2)	0.0462 (19)	0.047 (3)	−0.0027 (17)	0.0054 (19)	−0.0025 (18)
C41A	0.042 (2)	0.055 (2)	0.047 (2)	−0.0127 (19)	0.0044 (19)	−0.0013 (19)
C42A	0.052 (2)	0.057 (2)	0.053 (3)	−0.017 (2)	0.003 (2)	−0.007 (2)
Cl1	0.0790 (8)	0.0732 (7)	0.0875 (9)	0.0024 (6)	0.0178 (8)	−0.0225 (7)
C43A	0.070 (3)	0.076 (3)	0.056 (3)	−0.029 (3)	0.012 (2)	−0.011 (3)
C44A	0.068 (3)	0.089 (3)	0.054 (3)	−0.032 (3)	−0.011 (3)	0.018 (3)
Cl2	0.1222 (13)	0.1320 (11)	0.0591 (10)	−0.0232 (10)	−0.0130 (10)	0.0306 (9)
C45A	0.056 (3)	0.077 (3)	0.057 (3)	−0.003 (2)	−0.001 (3)	0.014 (2)
C46A	0.053 (2)	0.064 (2)	0.053 (3)	−0.003 (2)	0.006 (2)	0.004 (2)
C5A	0.0400 (19)	0.0478 (19)	0.054 (3)	−0.0066 (18)	0.002 (2)	0.0022 (19)
C6A	0.040 (2)	0.0335 (17)	0.051 (2)	−0.0023 (15)	0.0024 (18)	−0.0063 (17)
C61A	0.0406 (19)	0.0340 (17)	0.053 (2)	0.0003 (15)	0.0065 (19)	0.0057 (17)
C62A	0.048 (2)	0.0451 (19)	0.078 (4)	0.0075 (18)	−0.004 (3)	−0.006 (2)
C63A	0.056 (3)	0.061 (2)	0.069 (4)	0.004 (2)	−0.013 (3)	−0.005 (2)
C64A	0.035 (2)	0.066 (3)	0.087 (4)	0.005 (2)	−0.007 (2)	0.019 (3)
F64A	0.0455 (14)	0.114 (2)	0.137 (3)	0.0075 (15)	−0.0203 (17)	0.003 (2)
C65A	0.039 (2)	0.055 (2)	0.103 (4)	0.0128 (19)	0.012 (3)	0.005 (3)
C66A	0.049 (2)	0.0389 (19)	0.081 (3)	−0.0014 (18)	0.011 (2)	−0.009 (2)
C1B	0.0366 (18)	0.0299 (15)	0.037 (2)	0.0013 (14)	0.0045 (16)	−0.0012 (15)
C11B	0.042 (2)	0.0353 (18)	0.045 (2)	−0.0052 (16)	0.0034 (18)	0.0031 (16)
O12B	0.0744 (19)	0.0343 (14)	0.079 (2)	0.0055 (13)	0.0166 (18)	0.0016 (14)
O13B	0.0641 (17)	0.0537 (15)	0.0394 (15)	−0.0038 (14)	0.0094 (14)	0.0091 (13)
C14B	0.081 (3)	0.087 (3)	0.051 (3)	−0.009 (3)	0.011 (3)	0.031 (2)
C15B	0.080 (4)	0.129 (5)	0.109 (5)	−0.039 (4)	−0.002 (4)	0.047 (4)
C2B	0.036 (2)	0.055 (2)	0.049 (2)	−0.0001 (18)	0.0026 (19)	0.0060 (19)
O2B	0.0388 (15)	0.134 (3)	0.070 (2)	−0.0208 (18)	−0.0048 (15)	0.040 (2)
C3B	0.038 (2)	0.067 (2)	0.041 (3)	−0.0034 (19)	−0.0054 (19)	0.006 (2)
C4B	0.044 (2)	0.0414 (18)	0.034 (2)	−0.0045 (17)	−0.0003 (17)	−0.0007 (16)
C41B	0.044 (2)	0.0466 (18)	0.039 (2)	−0.0064 (17)	−0.0003 (18)	0.0050 (17)
C42B	0.050 (2)	0.0528 (19)	0.041 (2)	−0.0140 (19)	0.008 (2)	−0.003 (2)
Cl3	0.0777 (8)	0.0692 (6)	0.0624 (7)	0.0108 (6)	0.0090 (6)	−0.0134 (6)
C43B	0.059 (2)	0.084 (3)	0.035 (2)	−0.014 (2)	0.006 (2)	0.002 (2)
C44B	0.060 (3)	0.086 (3)	0.038 (3)	−0.011 (3)	−0.004 (2)	0.021 (2)
Cl4	0.0914 (10)	0.1618 (14)	0.0586 (10)	−0.0019 (10)	−0.0095 (8)	0.0507 (9)
C45B	0.058 (3)	0.073 (3)	0.068 (4)	0.005 (2)	−0.002 (3)	0.021 (3)
C46B	0.051 (2)	0.066 (2)	0.041 (2)	0.000 (2)	0.0036 (19)	0.009 (2)
C5B	0.0385 (19)	0.0465 (18)	0.035 (2)	−0.0061 (17)	0.0018 (17)	−0.0001 (17)
C6B	0.0351 (18)	0.0352 (16)	0.038 (2)	−0.0022 (15)	0.0057 (16)	−0.0082 (15)
C61B	0.0369 (17)	0.0279 (14)	0.042 (2)	0.0009 (14)	0.0040 (17)	0.0009 (15)
C62B	0.047 (2)	0.050 (2)	0.063 (3)	0.0185 (18)	−0.009 (2)	−0.019 (2)
C63B	0.049 (2)	0.063 (3)	0.076 (4)	0.000 (2)	−0.019 (3)	−0.023 (2)
C64B	0.037 (2)	0.063 (2)	0.069 (3)	0.006 (2)	−0.004 (2)	−0.001 (2)
F64B	0.0406 (13)	0.107 (2)	0.126 (3)	0.0133 (14)	−0.0186 (16)	−0.0152 (19)
C65B	0.042 (2)	0.053 (2)	0.097 (4)	0.0104 (19)	0.012 (3)	−0.007 (2)
C66B	0.040 (2)	0.0432 (19)	0.074 (3)	0.0001 (17)	0.013 (2)	−0.012 (2)

### Geometric parameters (Å, °)

**Table:**

C1A—C11A	1.501 (5)	C1B—C11B	1.503 (4)
C1A—C6A	1.518 (5)	C1B—C2B	1.520 (5)
C1A—C2A	1.525 (5)	C1B—C6B	1.547 (5)
C1A—H1A	0.9800	C1B—H1B	0.9800
C11A—O12A	1.193 (4)	C11B—O12B	1.194 (4)
C11A—O13A	1.326 (5)	C11B—O13B	1.326 (4)
O13A—C14A	1.470 (5)	O13B—C14B	1.453 (5)
C14A—C15A	1.431 (7)	C14B—C15B	1.456 (6)
C14A—H14B	0.9700	C14B—H14D	0.9700
C14A—H14A	0.9700	C14B—H14C	0.9700
C15A—H15C	0.9600	C15B—H15F	0.9600
C15A—H15B	0.9600	C15B—H15E	0.9600
C15A—H15A	0.9600	C15B—H15D	0.9600
C2A—O2A	1.220 (4)	C2B—O2B	1.227 (4)
C2A—C3A	1.435 (6)	C2B—C3B	1.452 (6)
C3A—C4A	1.348 (5)	C3B—C4B	1.318 (5)
C3A—H3A	0.9300	C3B—H3B	0.9300
C4A—C41A	1.473 (6)	C4B—C41B	1.488 (5)
C4A—C5A	1.503 (5)	C4B—C5B	1.506 (5)
C41A—C42A	1.384 (6)	C41B—C42B	1.384 (5)
C41A—C46A	1.403 (5)	C41B—C46B	1.387 (5)
C42A—C43A	1.373 (6)	C42B—C43B	1.378 (6)
C42A—Cl1	1.739 (4)	C42B—Cl3	1.742 (4)
C43A—C44A	1.398 (7)	C43B—C44B	1.359 (6)
C43A—H43A	0.9300	C43B—H43B	0.9300
C44A—C45A	1.373 (7)	C44B—C45B	1.365 (6)
C44A—Cl2	1.726 (5)	C44B—Cl4	1.740 (4)
C45A—C46A	1.374 (6)	C45B—C46B	1.390 (6)
C45A—H45A	0.9300	C45B—H45B	0.9300
C46A—H46A	0.9300	C46B—H46B	0.9300
C5A—C6A	1.528 (5)	C5B—C6B	1.525 (5)
C5A—H5A2	0.9700	C5B—H5B2	0.9700
C5A—H5A1	0.9700	C5B—H5B1	0.9700
C6A—C61A	1.518 (5)	C6B—C61B	1.510 (5)
C6A—H6A	0.9800	C6B—H6B	0.9800
C61A—C62A	1.367 (6)	C61B—C62B	1.379 (5)
C61A—C66A	1.384 (5)	C61B—C66B	1.381 (5)
C62A—C63A	1.384 (6)	C62B—C63B	1.397 (5)
C62A—H62A	0.9300	C62B—H62B	0.9300
C63A—C64A	1.359 (6)	C63B—C64B	1.359 (5)
C63A—H63A	0.9300	C63B—H63B	0.9300
C64A—C65A	1.347 (6)	C64B—C65B	1.354 (6)
C64A—F64A	1.363 (5)	C64B—F64B	1.359 (4)
C65A—C66A	1.388 (5)	C65B—C66B	1.383 (5)
C65A—H65A	0.9300	C65B—H65B	0.9300
C66A—H66A	0.9300	C66B—H66B	0.9300
			
C11A—C1A—C6A	113.9 (3)	C11B—C1B—C2B	106.8 (3)
C11A—C1A—C2A	106.9 (3)	C11B—C1B—C6B	113.5 (3)
C6A—C1A—C2A	110.8 (3)	C2B—C1B—C6B	110.4 (3)
C11A—C1A—H1A	108.4	C11B—C1B—H1B	108.6
C6A—C1A—H1A	108.4	C2B—C1B—H1B	108.6
C2A—C1A—H1A	108.4	C6B—C1B—H1B	108.6
O12A—C11A—O13A	124.1 (4)	O12B—C11B—O13B	124.4 (3)
O12A—C11A—C1A	124.4 (4)	O12B—C11B—C1B	123.5 (4)
O13A—C11A—C1A	111.5 (3)	O13B—C11B—C1B	112.1 (3)
C11A—O13A—C14A	117.3 (4)	C11B—O13B—C14B	116.7 (3)
C15A—C14A—O13A	111.6 (5)	O13B—C14B—C15B	112.2 (4)
C15A—C14A—H14B	109.3	O13B—C14B—H14D	109.2
O13A—C14A—H14B	109.3	C15B—C14B—H14D	109.2
C15A—C14A—H14A	109.3	O13B—C14B—H14C	109.2
O13A—C14A—H14A	109.3	C15B—C14B—H14C	109.2
H14B—C14A—H14A	108.0	H14D—C14B—H14C	107.9
C14A—C15A—H15C	109.5	C14B—C15B—H15F	109.5
C14A—C15A—H15B	109.5	C14B—C15B—H15E	109.5
H15C—C15A—H15B	109.5	H15F—C15B—H15E	109.5
C14A—C15A—H15A	109.5	C14B—C15B—H15D	109.5
H15C—C15A—H15A	109.5	H15F—C15B—H15D	109.5
H15B—C15A—H15A	109.5	H15E—C15B—H15D	109.5
O2A—C2A—C3A	121.2 (4)	O2B—C2B—C3B	122.0 (4)
O2A—C2A—C1A	121.3 (4)	O2B—C2B—C1B	120.6 (4)
C3A—C2A—C1A	117.5 (3)	C3B—C2B—C1B	117.4 (3)
C4A—C3A—C2A	123.4 (4)	C4B—C3B—C2B	123.5 (4)
C4A—C3A—H3A	118.3	C4B—C3B—H3B	118.3
C2A—C3A—H3A	118.3	C2B—C3B—H3B	118.3
C3A—C4A—C41A	119.9 (4)	C3B—C4B—C41B	118.7 (4)
C3A—C4A—C5A	119.9 (4)	C3B—C4B—C5B	120.9 (4)
C41A—C4A—C5A	120.2 (3)	C41B—C4B—C5B	120.3 (3)
C42A—C41A—C46A	117.0 (4)	C42B—C41B—C46B	117.5 (4)
C42A—C41A—C4A	123.7 (4)	C42B—C41B—C4B	123.3 (3)
C46A—C41A—C4A	119.2 (4)	C46B—C41B—C4B	119.1 (4)
C43A—C42A—C41A	122.3 (4)	C43B—C42B—C41B	121.8 (4)
C43A—C42A—Cl1	117.8 (4)	C43B—C42B—Cl3	117.6 (3)
C41A—C42A—Cl1	119.8 (3)	C41B—C42B—Cl3	120.6 (3)
C42A—C43A—C44A	118.5 (4)	C44B—C43B—C42B	118.8 (4)
C42A—C43A—H43A	120.8	C44B—C43B—H43B	120.6
C44A—C43A—H43A	120.8	C42B—C43B—H43B	120.6
C45A—C44A—C43A	121.4 (4)	C43B—C44B—C45B	122.1 (4)
C45A—C44A—Cl2	119.8 (4)	C43B—C44B—Cl4	118.7 (4)
C43A—C44A—Cl2	118.7 (4)	C45B—C44B—Cl4	119.2 (4)
C44A—C45A—C46A	118.4 (4)	C44B—C45B—C46B	118.5 (4)
C44A—C45A—H45A	120.8	C44B—C45B—H45B	120.8
C46A—C45A—H45A	120.8	C46B—C45B—H45B	120.8
C45A—C46A—C41A	122.4 (4)	C41B—C46B—C45B	121.3 (4)
C45A—C46A—H46A	118.8	C41B—C46B—H46B	119.3
C41A—C46A—H46A	118.8	C45B—C46B—H46B	119.3
C4A—C5A—C6A	112.4 (3)	C4B—C5B—C6B	112.0 (3)
C4A—C5A—H5A2	109.1	C4B—C5B—H5B2	109.2
C6A—C5A—H5A2	109.1	C6B—C5B—H5B2	109.2
C4A—C5A—H5A1	109.1	C4B—C5B—H5B1	109.2
C6A—C5A—H5A1	109.1	C6B—C5B—H5B1	109.2
H5A2—C5A—H5A1	107.8	H5B2—C5B—H5B1	107.9
C1A—C6A—C61A	114.9 (3)	C61B—C6B—C5B	111.2 (3)
C1A—C6A—C5A	108.6 (3)	C61B—C6B—C1B	114.4 (3)
C61A—C6A—C5A	111.2 (3)	C5B—C6B—C1B	108.3 (3)
C1A—C6A—H6A	107.3	C61B—C6B—H6B	107.6
C61A—C6A—H6A	107.3	C5B—C6B—H6B	107.6
C5A—C6A—H6A	107.3	C1B—C6B—H6B	107.6
C62A—C61A—C66A	117.4 (4)	C62B—C61B—C66B	117.1 (3)
C62A—C61A—C6A	122.3 (3)	C62B—C61B—C6B	122.3 (3)
C66A—C61A—C6A	120.2 (3)	C66B—C61B—C6B	120.5 (3)
C61A—C62A—C63A	121.4 (4)	C61B—C62B—C63B	121.4 (3)
C61A—C62A—H62A	119.3	C61B—C62B—H62B	119.3
C63A—C62A—H62A	119.3	C63B—C62B—H62B	119.3
C64A—C63A—C62A	119.1 (4)	C64B—C63B—C62B	118.7 (4)
C64A—C63A—H63A	120.5	C64B—C63B—H63B	120.6
C62A—C63A—H63A	120.5	C62B—C63B—H63B	120.6
C65A—C64A—C63A	122.0 (4)	C65B—C64B—F64B	119.5 (4)
C65A—C64A—F64A	118.8 (4)	C65B—C64B—C63B	121.9 (4)
C63A—C64A—F64A	119.3 (4)	F64B—C64B—C63B	118.6 (4)
C64A—C65A—C66A	118.3 (4)	C64B—C65B—C66B	118.7 (4)
C64A—C65A—H65A	120.9	C64B—C65B—H65B	120.6
C66A—C65A—H65A	120.9	C66B—C65B—H65B	120.6
C61A—C66A—C65A	121.8 (4)	C61B—C66B—C65B	122.2 (4)
C61A—C66A—H66A	119.1	C61B—C66B—H66B	118.9
C65A—C66A—H66A	119.1	C65B—C66B—H66B	118.9
			
C6A—C1A—C11A—O12A	−31.8 (5)	C2B—C1B—C11B—O12B	−88.3 (4)
C2A—C1A—C11A—O12A	90.9 (4)	C6B—C1B—C11B—O12B	33.7 (5)
C6A—C1A—C11A—O13A	149.9 (3)	C2B—C1B—C11B—O13B	88.6 (3)
C2A—C1A—C11A—O13A	−87.4 (4)	C6B—C1B—C11B—O13B	−149.4 (3)
O12A—C11A—O13A—C14A	−8.7 (6)	O12B—C11B—O13B—C14B	7.7 (6)
C1A—C11A—O13A—C14A	169.6 (3)	C1B—C11B—O13B—C14B	−169.1 (3)
C11A—O13A—C14A—C15A	−87.8 (6)	C11B—O13B—C14B—C15B	85.0 (5)
C11A—C1A—C2A—O2A	21.0 (5)	C11B—C1B—C2B—O2B	−22.8 (5)
C6A—C1A—C2A—O2A	145.6 (4)	C6B—C1B—C2B—O2B	−146.7 (4)
C11A—C1A—C2A—C3A	−157.5 (3)	C11B—C1B—C2B—C3B	156.0 (3)
C6A—C1A—C2A—C3A	−32.9 (4)	C6B—C1B—C2B—C3B	32.1 (4)
O2A—C2A—C3A—C4A	−173.7 (4)	O2B—C2B—C3B—C4B	175.2 (4)
C1A—C2A—C3A—C4A	4.8 (6)	C1B—C2B—C3B—C4B	−3.5 (6)
C2A—C3A—C4A—C41A	179.8 (4)	C2B—C3B—C4B—C41B	179.8 (3)
C2A—C3A—C4A—C5A	−2.0 (6)	C2B—C3B—C4B—C5B	0.8 (6)
C3A—C4A—C41A—C42A	−119.3 (4)	C3B—C4B—C41B—C42B	117.8 (4)
C5A—C4A—C41A—C42A	62.6 (5)	C5B—C4B—C41B—C42B	−63.2 (5)
C3A—C4A—C41A—C46A	56.6 (5)	C3B—C4B—C41B—C46B	−58.8 (5)
C5A—C4A—C41A—C46A	−121.6 (4)	C5B—C4B—C41B—C46B	120.2 (4)
C46A—C41A—C42A—C43A	−0.5 (6)	C46B—C41B—C42B—C43B	0.0 (6)
C4A—C41A—C42A—C43A	175.5 (4)	C4B—C41B—C42B—C43B	−176.6 (3)
C46A—C41A—C42A—Cl1	−176.6 (3)	C46B—C41B—C42B—Cl3	178.6 (3)
C4A—C41A—C42A—Cl1	−0.6 (5)	C4B—C41B—C42B—Cl3	1.9 (5)
C41A—C42A—C43A—C44A	2.1 (6)	C41B—C42B—C43B—C44B	−0.5 (6)
Cl1—C42A—C43A—C44A	178.3 (3)	Cl3—C42B—C43B—C44B	−179.1 (3)
C42A—C43A—C44A—C45A	−1.5 (6)	C42B—C43B—C44B—C45B	0.4 (6)
C42A—C43A—C44A—Cl2	176.8 (3)	C42B—C43B—C44B—Cl4	179.8 (3)
C43A—C44A—C45A—C46A	−0.7 (7)	C43B—C44B—C45B—C46B	0.3 (7)
Cl2—C44A—C45A—C46A	−179.0 (3)	Cl4—C44B—C45B—C46B	−179.1 (3)
C44A—C45A—C46A—C41A	2.4 (6)	C42B—C41B—C46B—C45B	0.7 (6)
C42A—C41A—C46A—C45A	−1.9 (6)	C4B—C41B—C46B—C45B	177.4 (4)
C4A—C41A—C46A—C45A	−178.0 (4)	C44B—C45B—C46B—C41B	−0.8 (6)
C3A—C4A—C5A—C6A	27.7 (5)	C3B—C4B—C5B—C6B	−27.6 (5)
C41A—C4A—C5A—C6A	−154.1 (3)	C41B—C4B—C5B—C6B	153.4 (3)
C11A—C1A—C6A—C61A	−57.8 (4)	C4B—C5B—C6B—C61B	−179.0 (3)
C2A—C1A—C6A—C61A	−178.3 (3)	C4B—C5B—C6B—C1B	54.4 (4)
C11A—C1A—C6A—C5A	177.1 (3)	C11B—C1B—C6B—C61B	59.0 (4)
C2A—C1A—C6A—C5A	56.5 (4)	C2B—C1B—C6B—C61B	178.9 (3)
C4A—C5A—C6A—C1A	−54.5 (4)	C11B—C1B—C6B—C5B	−176.4 (3)
C4A—C5A—C6A—C61A	178.2 (3)	C2B—C1B—C6B—C5B	−56.5 (3)
C1A—C6A—C61A—C62A	−55.0 (5)	C5B—C6B—C61B—C62B	−67.1 (5)
C5A—C6A—C61A—C62A	68.8 (5)	C1B—C6B—C61B—C62B	56.0 (5)
C1A—C6A—C61A—C66A	127.1 (4)	C5B—C6B—C61B—C66B	109.6 (4)
C5A—C6A—C61A—C66A	−109.1 (4)	C1B—C6B—C61B—C66B	−127.4 (3)
C66A—C61A—C62A—C63A	−0.5 (7)	C66B—C61B—C62B—C63B	−2.1 (7)
C6A—C61A—C62A—C63A	−178.4 (4)	C6B—C61B—C62B—C63B	174.7 (4)
C61A—C62A—C63A—C64A	1.1 (7)	C61B—C62B—C63B—C64B	2.0 (8)
C62A—C63A—C64A—C65A	−1.4 (7)	C62B—C63B—C64B—C65B	−1.0 (8)
C62A—C63A—C64A—F64A	178.6 (4)	C62B—C63B—C64B—F64B	−179.4 (5)
C63A—C64A—C65A—C66A	1.1 (7)	F64B—C64B—C65B—C66B	178.7 (4)
F64A—C64A—C65A—C66A	−178.9 (4)	C63B—C64B—C65B—C66B	0.3 (7)
C62A—C61A—C66A—C65A	0.1 (6)	C62B—C61B—C66B—C65B	1.4 (6)
C6A—C61A—C66A—C65A	178.1 (4)	C6B—C61B—C66B—C65B	−175.5 (4)
C64A—C65A—C66A—C61A	−0.4 (7)	C64B—C65B—C66B—C61B	−0.4 (7)

### Hydrogen-bond geometry (Å, °)

**Table:**

*D*—H···*A*	*D*—H	H···*A*	*D*···*A*	*D*—H···*A*
C1A—H1A···O12A^i^	0.98	2.40	3.306 (4)	154
C62A—H62A···O12A^i^	0.93	2.55	3.442 (5)	162
C1B—H1B···O12B^ii^	0.98	2.39	3.292 (4)	153
C62B—H62B···O12B^ii^	0.93	2.47	3.358 (4)	161
C14A—H14A···Cl1^iii^	0.97	2.82	3.725 (6)	155
C3A—H3A···O2B	0.93	2.54	3.360 (5)	148
C3B—H3B···O2A	0.93	2.51	3.352 (5)	151

Symmetry codes: (i) *x*, *y*+1, *z*; (ii) *x*, *y*−1, *z*; (iii) −*x*+1/2, *y*, *z*+1/2.
